# Matrix Metalloproteinase-9 (MMP9)—A Mediating Enzyme in Cardiovascular Disease, Cancer, and Neuropsychiatric Disorders

**DOI:** 10.1155/2009/904836

**Published:** 2009-08-31

**Authors:** Janusz K. Rybakowski

**Affiliations:** Department of Adult Psychiatry, Poznan University of Medical Sciences, ul.Szpitalna 27/33, 60-572 Poznan, Poland

## Abstract

Matrix metalloproteinase-9 (MMP9) has been implicated in numerous somatic illnesses, including cardiovascular disorders and cancer. Recently, MMP9 has been shown to be increasingly important in several aspects of central nervous system activity. Furthermore, a pathogenic role for this enzyme has been suggested in such neuropsychiatric disorders as schizophrenia, bipolar illness, and multiple sclerosis. In this paper, the results of biochemical and molecular-genetic studies on MMP9 that have been performed in these pathological conditions will be summarized. Furthermore, I hypothesize that the *MMP9* gene, as shown by functional −1562 C/T polymorphism studies, may be mediating the relationship of neuropsychiatric illnesses (schizophrenia, bipolar mood disorder, multiple sclerosis) that are comorbid with cardiovascular disease and cancer.

## 1. Introduction

 The matrix metalloproteinases (MMPs) are a large family of zinc-dependent, extracellularly acting endopeptidases, the substrates of which are proteins of the extracellular matrix and adhesion proteins [1]. Matrix metalloproteinase-9 (MMP9), also known as gelatinase B, 92 kDa gelatinase, or 92 Da type IV collagenase (which represents the largest and most complex member of this family) has recently been a subject of growing interest in human pathology. 

In recent years, MMPs have attracted interest as mediators of both pathology and regeneration in the central nervous system [2]. Concerning MMP9, a role for this enzyme in the plasticity of the central nervous system has been investigated in experimental studies [3]. Blocking of MMP9, either by pharmacological or genetic means, selectively inhibits hippocampal late-phase long-term potentiation as well as fear memory in mice [4]. Furthermore, TIMP-1, the endogenous inhibitor of MMP9, abolished MMP9-dependent long-term potentiation in the prefrontal cortex of freely moving rats [5]. In addition, a pathogenic role has been proposed for MMP9 in an animal model of aberrant plasticity [6] and temporal lobe epilepsy [7].

The human MMP9 gene was mapped to the chromosome region 20q11.2–q13.1 [8] and several polymorphisms of this gene were identified. The −1562 C/T polymorphism (rs3918242) was shown to exert a functional effect on gene transcription. This single nucleotide polymorphism (SNP) at −1562 bp is due to a C to T substitution (−1562 C→T), which results in the loss of binding of a nuclear protein to this region and an increase in transcriptional activity in macrophages. In these cells, the C/C genotype leads to a low promoter activity whereas the C/T and T/T genotypes result in high transcriptional activity [9]. 

The molecular-genetic studies of the functional −1562 C/T polymorphism of the *MMP9* gene brought about interesting results in cardiovascular, cancer, and neuropsychiatric conditions. Research in cardiovascular illness and cancer showed that carriers of the T allele have an increased severity of coronary arteriosclerosis [10], increased cardiac mortality [11], and increased risk or more severe progression of some types of cancer [12, 13]. Recent studies also demonstrated an association of this polymorphism with a predisposition to schizophrenia [14], bipolar illness [15], and multiple sclerosis [16, 17].

Based on the results of these studies, I hypothesize that the *MMP9* gene, which has a functional −1562 C/T polymorphism, may mediate the epidemiological comorbidity of neuropsychiatric illnesses (schizophrenia, bipolar mood disorder, multiple sclerosis) with cardiovascular diseases and cancer. 

## 2. MMP-9 in Cardiovascular Disease

In a large prospective study of middle-aged men (465 cases, 1076 controls), Welsh et al. [18] showed an association of serum MMP9 with the incidence of coronary heart disease in the general population. More detailed studies have recently been performed in middle-aged population by Swedish researchers who found an association of circulating MMP9 levels not only with cardiovascular [19] but also with psychosocial risk factors for coronary artery disease (e.g., depression) [20]. Related to these observations, an inverse relationship between markers of nitric oxide formation and MMP9 was found in healthy subjects [21]. The higher level of MMP9 in patients with coronary artery disease has been recently reported [22]. Higher MMP9 level was also a correlate of coronary artery ectasia [23] and a predictor of increased mortality in patients with coronary artery disease [24].

The association of MMP9 status with a progression of coronary heart disease has also been confirmed by molecular genetic studies that used the functional −1562 C/T polymorphism of MMP9 gene. It was observed that the carriers of the T allele had increased cardiac mortality [11], and more recently, an association of T allele with myocardial infarction in patients with coronary heart disease was found [25]. In cardiac patients, a relationship was also demonstrated between the T allele of the −1562 C/T polymorphism and markedly increased levels of MMP9 [24], compatible with higher transcriptional activity of this allele in experimental studies [9]. A similar relationship between plasma MMP9 and the T allele of the −1562 C/T polymorphism was also shown in HIV patients under antiviral therapy [26].

Recently, Konstantino et al. [27] pointed out the prominent role of MMP9 in plaque formation, destabilization, and rupture, and postulated that MMP9 levels may serve as a biomarker for acute coronary syndrome. An association of MMP9 levels with atherosclerotic changes has been previously found in patients with atherosclerosis of the femoral artery [28] and with chronic periodontitis [29]. Higher levels of MMP9 were also observed in hypertrophic cardiomyopathy, which correlated with a worse prognosis [30]. In molecular-genetic studies that genotyped the functional −1562 C/T polymorphism of the MMP9 gene, it was observed that the carriers of the T allele had an increased severity of coronary atherosclerosis [10].

The available data also show a possible association of MMP9 with the pathogenesis and treatment of hypertensive disease. Higher MMP9 level were found preclinically in spontaneously hypertensive hyperlipidemic rats [31] and clinically in women with gestational hypertension [32]. In the group of 595 patients evaluated in the Framingham Offspring Study, higher MMP9 concentrations were related to higher risk of blood pressure progression [33]. Recently, it was demonstrated that plasma MMP9 samples were inhibited by captopril to a similar extent as the angiotensin-converting enzyme [34]. 

## 3. MMP-9 in Cancer

Sakata et al. [35] showed an overexpression of MMP9 in an epithelial tumor of the ovary and its contribution to lymph node metastases of ovarian carcinoma cells. Similarly, in patients with breast cancer, increased serum and tissue expression of MMP9 was associated with a worse prognosis of the course of tumor [36]. Higher levels of MMP9 have been reported in endometrial polyps, especially in those occurring in premenopausal women [37]. Recently, higher MMP9 levels were also observed in pulmonary lymphangio-leiomyomatosis characterized by excessive cell proliferation [38].

Molecular-genetic studies of the functional −1562 C/T polymorphism of the MMP9 gene have revealed a frequent association of T allele with an increased risk of some kinds of cancer and with more severe progression of the tumor and/or greater dynamics of metastases. Sugimoto et al. [12] observed that the T allele was associated with endometrial carcinoma risk in a Japanese population. Other studies showed an association of the T allele with the risk for oral squamous cell carcinoma in younger male areca users [39] and with genetic risk for esophageal squamous cell carcinoma [40]. Kader et al. [41] demonstrated that several MMP9 haplotypes (including −1562 C/T polymorphism) were associated with the risk of invasive cancer of the urinary bladder. Concerning gastric cancer, it has been found that the T allele of the −1562 C/T polymorphism of MMP9 gene is associated with an invasive phenotype of this tumor [42] and with a higher frequency of lymph node metastasis [43]. In breast cancer, Przybylowska et al. [44] reported that the T allele of this polymorphism was associated with malignance and growth of tumors, and Hughes et al. [13] showed an association with the severity of lymph node metastases. Higher risk of lymph node metastases in colorectal cancer was also found to be connected with the T allele [45].

## 4. MMP-9 in Multiple Sclerosis

An upregulation of MMPs with a decrease of tissue inhibitors (TIMPs) in biological fluids of multiple sclerosis (MS) patients and in an animal model of the disease has been found in numerous studies. Further, the potential of drugs affecting MMPs for treatment of MS has been discussed [46]. A significant elevation of MMP9 related to various courses of MS has been found [47]. Also recently, Shinto et al. [48] demonstrated that omega-3 fatty acid supplementation decreased MMP9 levels in relapsing-remitting MS.

In recent years, molecular-genetic studies have focused on the functional −1562 C/T polymorphism of the MMP9 gene in MS. In the first study performed in Serbia, it was found that T allele carriers had a lower susceptibility and severity of MS, and the T allele was found significantly less frequently in women with MS [16]. The second study performed in the Czech Republic confirmed these findings, showing a significant decrease of T allele in patients with MS compared to healthy subjects, especially females [17]. 

Recently, epidemiological studies investigating the comorbidity of MS and vascular disease and cancer were published. The first study was performed on 9949 hospitalizations of MS patients in New York City from 1988 through 2002. It was found that MS patients were less likely to be hospitalized for ischemic heart disease and myocardial infarction. However, they were more likely to be hospitalized for ischemic stroke than matched controls (general non-MS population) [49]. A second study performed in Sweden estimated cancer risk among 20 276 patients with MS and 203 951 individuals without MS using Swedish general population register data. In patients with MS, there was a decreased overall cancer risk, however, an increased risk for brain tumors was observed [50]. 

## 5. MMP-9 in Schizophrenia

Studies on the MMP9 levels in schizophrenia have not yet been performed. To investigate the *MMP9* gene in this illness, we genotyped the functional −1562 C/T polymorphism in a group of 442 schizophrenic patients and in 558 healthy control subjects. Since MMP9 influences hippocampal and prefrontal cortical activity [4, 5], we hypothesized that a polymorphism of the *MMP9* gene is associated with the pathogenesis of schizophrenia, a condition in which prefrontal cortex impairment is one of the most common pathological findings [51]. A significant preponderance of the C/C genotype and C allele, and the diminished frequency of the T allele of the −1562 polymorphism was found in schizophrenia subjects compared to healthy controls [14].

As shown previously, in both cardiovasular disease and cancer, T allele carriers present more severe pathological manifestations of these conditions [10–13]. Although the risk of cardiovascular disease in schizophrenia is reported to be similar to that of the general population [52], some studies show a more benign course of cardiovascular illness in such patients [53]. Also, compatible with our findings, a lower predisposition to cancer in schizophrenic patients has long been postulated [54], and the results of some recent analyses may partially favor such a concept [55, 56].

## 6. MMP-9 in Bipolar Mood Disorder

Similar to schizophrenia, there are no studies measuring MMP9 blood levels in patients with bipolar disorder. To investigate the status of the *MMP9* gene in this illness, we genotyped the functional −1562 C/T polymorphism in a group of 416 patients with bipolar mood disorder, including 75 patients with bipolar type II, and in 558 healthy control subjects. This approach has been substantiated by previous reports on the significance of MMP9 for hippocampal and prefrontal cortical activity and for aspects of brain functions such as neuroplasticity and epileptogenesis [4–7]. Patients with bipolar mood disorder had a significant preponderance of T allele versus C allele of the −1562 C/T polymorphism of the *MMP9* gene compared to healthy control subjects. The higher frequency of the T allele was especially evident in a subgroup of patients with bipolar disorder type II compared to healthy subjects [15].

Compatible with the finding that T allele carriers present more severe pathological manifestations of cardiovascular disease and cancer [10–13] are findings from a recent epidemiological study demonstraing an enhanced cancer risk among patients with bipolar disorder [57]. A Swedish epidemiological study also showed more than a 2.5-fold increased mortality rate from cardiovascular disease in bipolar patients [58]. 

## 7. MMP-9 and Neuropsychological Tests

In view of the experimental studies showing an involvement of MMP9 in prefrontal cortex functions in rats [5], we also performed neuropsychological tests measuring this activity in patients with schizophrenia and bipolar illness, and in control subjects in relation to −1562 C/T polymorphism of MMP9 gene. 173 patients with schizophrenia (89 male, 84 female), mean age 29 years, 177 patients with bipolar illness (68 male and 109 female), mean age 43 years, and 181 healthy subjects (86 male and 95 female), mean age 35 years, were included. For cognitive assessment, a computer version of the Wisconsin Card Sorting Test (WCST) was employed, with five domains reflecting working memory and executive functions, depending primarily on prefrontal cortex activity. Additionally, the Trail Making Test, A and B, and the Stroop test, A and B, were used.

In schizophrenia patients, no differences were found regarding neuropsychological performance among patients with various genotypes of the polymorphism (data not published). Among male patients with bipolar illness, the results for C/C homozygotes (*n* = 50) were better on all domains of the WCST compared with the remaining genotypes (*n* = 18): no differences were found in female patients. Bipolar males and females did not differ in mean age (43 + 15 years and 44 + 14 years) or mean duration of illness (12 + 12 years and 14 + 11 years, resp.). In males, the mean age and mean duration of the illness of C/C homozygotes were similar to patients with the remaining genotypes [59].

In the only previous study measuring the impact of MMP9 gene on cognitive functions, Vassos et al. [60] found no association between hippocampus-dependent episodic memory and functional repeat polymorphism (CA)n of the *MMP9* gene in healthy subjects. Also, in control subjects studied by us, comparison of cognitive test results within genotypes did not reveal significant differences either in the whole group or in male and female patients. The only difference was in Stroop test, part A, in male patients, where the results for C/C homozygotes (*n* = 66) were better than other genotypes combined (*n* = 20). This difference in performance related to genotypes was similar to that obtained in male bipolar patients on WCST domains. Healthy male and female subjects did not differ in mean age (34 ± 11 years and 36 ± 12 years, resp.) [61].

These results suggest that in humans, neuropsychological functions and MMP9 enzyme activity may not have a direct correlation. Thus, increased activity of the MMP9 system was associated with higher levels of prefrontal function in experimental animals models [4, 5], also with neuropsychiatric illnesses such as schizophrenia or multiple sclerosis [16, 17] and The results obtained in males with bipolar illness on the WCST and in healthy males on the Stroop test may suggest that under certain conditions, a correlation of higher levels of neuropsychological function with C allele (connected with lower transcriptional activity for the MMP9 gene) may exist. 

## 8. Matrix Metalloproteinase-9 (MMP-9)—A Putative Mediating Enzyme for Cardiovascular Disorder, Cancer, and Neuropsychiatric Disorders

**Figure 1:**
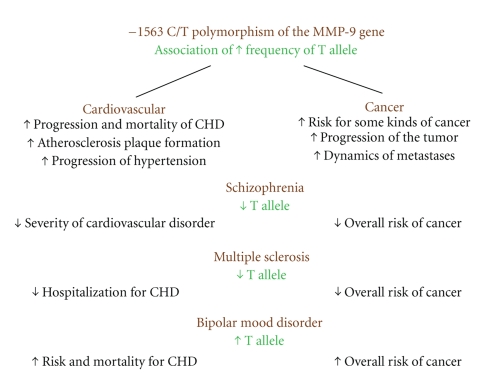
Epidemiological relationships between cardiovascular disorder, cancer, schizophrenia, multiple sclerosis, and bipolar mood disorder and the functional −1562 polymorphism of the *MMP9* gene.

Because of the functional implications of the −1562 C/T polymorphism of the *MMP9* gene, the comorbidity of cardiovascular disorders, cancer, and such neuropsychiatric illnesses as schizophrenia, bipolar mood disorder, and multiple sclerosis can be hypothesized (Figure 1).

Hence, the T allele of the −1562 polymorphism of MMP9 gene is related to a higher transcriptional activity of the gene and in cardiovascular illness and cancer to higher MMP-levels in biological fluids and tissues. In cardiovascular illness, carrying of the T allele and/or higher MMP9 levels are related to an increased progression and mortality of coronary heart disease (CHD) [25] increased atherosclerosis [10], and increased progression of hypertension [33]. Interestingly, in neuropsychiatric disorders with a lower frequency of the T allele, some epidemiological studies suggest a more benign course of cardiovascular disease, for example, in schizophrenia [53], and fewer cardiovascular hospitalizations in MS [49]. On the other hand, the phenomenon of higher risk for cardiovascular illness and higher mortality in patients with mood disorders (which have a higher frequency of the T allel carriers) has long been observed [58]. The proposed mediating factors include impairment in endothelial function that was demonstrated both in bipolar and unipolar depression [62] and, as hypothesized here, possibility the MMP9 system.

In oncology, the carrying of the T allele of the −1562 C/T MMP9 gene polymorphism is related to an increased risk for some kinds of cancer [12], more severe progression of tumor growth [42], and higher dynamics of metastases [45]. In neuropsychiatric disorders, some epidemiological studies suggest a lower overall incidence of cancer in schizophrenia [56] and in MS [50] (both illnesses with a lower frequency of T allele carriers), and increased cancer morbidity in bipolar mood disorder [57]. Interestingly, an association between bipolar mood disorder and cancer has been also found with respect to the levels of another metalloprotease, ADAM12 (a disintegrin and metalloprotease) [63, 64]. 

Nevertheless, it should be emphasized that in the central nervous system, there is more complex regulation of MMPs. As Agrawal et al. [65] pointed out “the good guys may go bad” under some conditions. There are several limitations to this hypothesis. The majority of referred molecular genetic research was performed with −1562 C/T functional polymorphisms of MMP9 but the other polymorphisms have not been sufficiently studied. Literature data on the human blood levels of MMPs used to develop this hypothesis were not evaluated for possible methodological issues [66]. Also, it should be acknowledged that there is a complex interplay of the *MMP9* gene with the other genes and environmental factors of MMPs family and with a host of other genes and with factors. However, it is conceivable that the *MMP9* gene is a mediating factor among cardiovascular disorders, cancer, schizophrenia, bipolar mood disorder, and multiple sclerosis. This is may contribute to a better explanation of the comorbidity between some somatic and neuropsychiatric illnesses.
